# A clinical case of successful palliative endovascular treatment of a patient with a single ventricle, mitral valve atresia, an intact atrial septum and persistent cardinal vein

**DOI:** 10.1186/s43044-023-00368-z

**Published:** 2023-05-18

**Authors:** Gorbatykh Artem, Manannikov Denis, Ivanilova Alina, Averkin Igor’, Zubarev Dmitriy, Prokhorikhin Aleksei, Soynov Ilya, Grekhov Evgeniy, Chernyavskiy Mikhail

**Affiliations:** 1 Almazov National Medical Research Centre, Akkuratova Str. 2, Saint-Petersburg, Russian Federation 197341; 2 E.N. Meshalkin National Medical Research Centre, Rechkunovskaya Str. 15, Novosibirsk, Russian Federation 630055

**Keywords:** Anomalous venous return, Endovascular palliative intervention

## Abstract

**Background:**

Treatment of newborns with univentricular hemodynamics in combination with an anomaly of pulmonary venous return has the worst correction results in modern cardiac surgical papers. According to the data obtained by different authors, postoperative mortality in this cohort of patients varies from 41.7 to 53%. The presence of the venous outflow tract obstruction, as well as the serious condition of a newborn, is one of the main factors that increase the risk of death in the postoperative period.

**Case presentation:**

This article reveals a clinical case of a patient with a combined heart disease prenatally diagnosed in the form of a functionally single ventricle with a double outlet of the main vessels from it, mitral valve atresia, an intact atrial septum and an anomaly of venous return, when the blood outflow from the left atrium was carried out through a single fetal communication such as stenotic cardinal vein. In order to stabilize the patient's condition, the newborn urgently underwent stenting of the stenotic section of the cardinal vein. However, due to the lack of positive dynamics in the postoperative period, the child underwent repeated endovascular intervention and stenting of the intraoperatively created interatrial communication was performed. Taking into account the absence of obstruction of the outflow tract to the pulmonary artery, it was necessary to perform an open surgical intervention in a short time such as pulmonary artery banding.

**Conclusions:**

Thus, palliative endovascular intervention in critically ill neonates with univentricular hemodynamics and anomalous pulmonary venous return can be considered as a method of choice that can become a new safer strategy for managing infants in order to stabilize the condition before the main stage of surgical intervention comes.

## Background

The congenital pathology of pulmonary venous return comprises a large and heterogeneous group of congenital heart defects (CHDs). Pulmonary venous return can be either an isolated vascular malformation or one aspect of a complicated CHD. Violation of pulmonary venous blood flow is of great importance in terms of the clinical course of the disease and its prognosis. Total anomalous pulmonary venous connection (TAPVC) is a classic example of a congenital pathology of pulmonary venous return when there is no direct drainage of the pulmonary veins into the left atrium, and pulmonary venous return is conducted through preserved communications with primitive cardinal and umbilical cord-vitelline outflow tracts [1, 2]. Another example of a congenital pulmonary venous (PV) outflow disorder is the combination of left heart hypoplasia with mitral valve (MV) atresia and an intact atrial septum (AS). In this case, while the correct connection between the pulmonary veins and the left atrium is maintained, venous outflow from the lungs is sharply limited, clinically similar to the effect of the obstructive form of TAPVC. Severe obstruction of the venous return pathways and intact atrial septum are indications for urgent surgical treatment due to the high risk of adverse outcomes [3, 4]. One treatment method for such patients is an endovascular intervention with balloon dilatation of stenotic vessels, through which pulmonary venous return occurs, followed by the possible implantation of a stent in the area of ​​stenosis [5, 6]. In this article, we present a clinical case of transcatheter treatment of obstructive pulmonary venous blood flow in a newborn with a functionally single ventricle with a double outlet of the main vessels, MV atresia, an intact AS and an anomaly of venous return, in which the outflow of blood from the left atrium was channeled through persistent fetal communication, i.e., the stenotic cardinal vein (CV).

## Case presentation

**Fig. 1:**
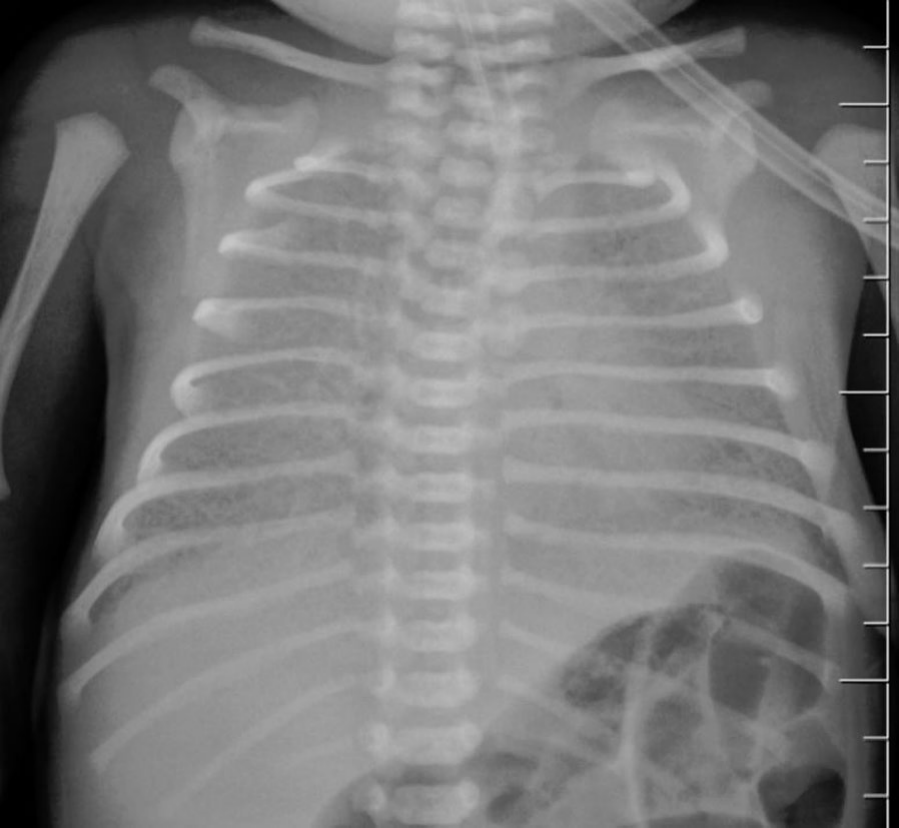
Chest X-ray, front view. Total infiltration of both lungs is determined mainly due to the compaction of the pulmonary interstitium

A CHD was suspected at a fetal ultrasound screening. The heart presented as a functionally single ventricle (SV), MV atresia and a double outlet right ventricle (DORV). A boy weighing 2,850 g was born by cesarean section at 38 weeks of gestation. The newborn’s condition from the first minutes of life was regarded as critical, and diffuse cyanosis of the skin was noted (saturation was 65–75%). The prenatal diagnosis was confirmed by echocardiograph (ECHO), and an intact AS was additionally detected. The pulmonary veins drained into the left atrium, and the outflow of blood was channeled via fetal communication through the persistent cardinal vein, into the left innominate vein (IV), and then into the superior vena cava (SVC). Additionally, cardinal vein stenosis with a narrowing of up to 1.5 mm and a blood flow velocity of 2.4–2.9 m/s (peak gradient is 27 mmHg) was revealed. The chest X-ray revealed signs of severe stagnation in the lungs (Fig. 1).

**Fig. 2:**
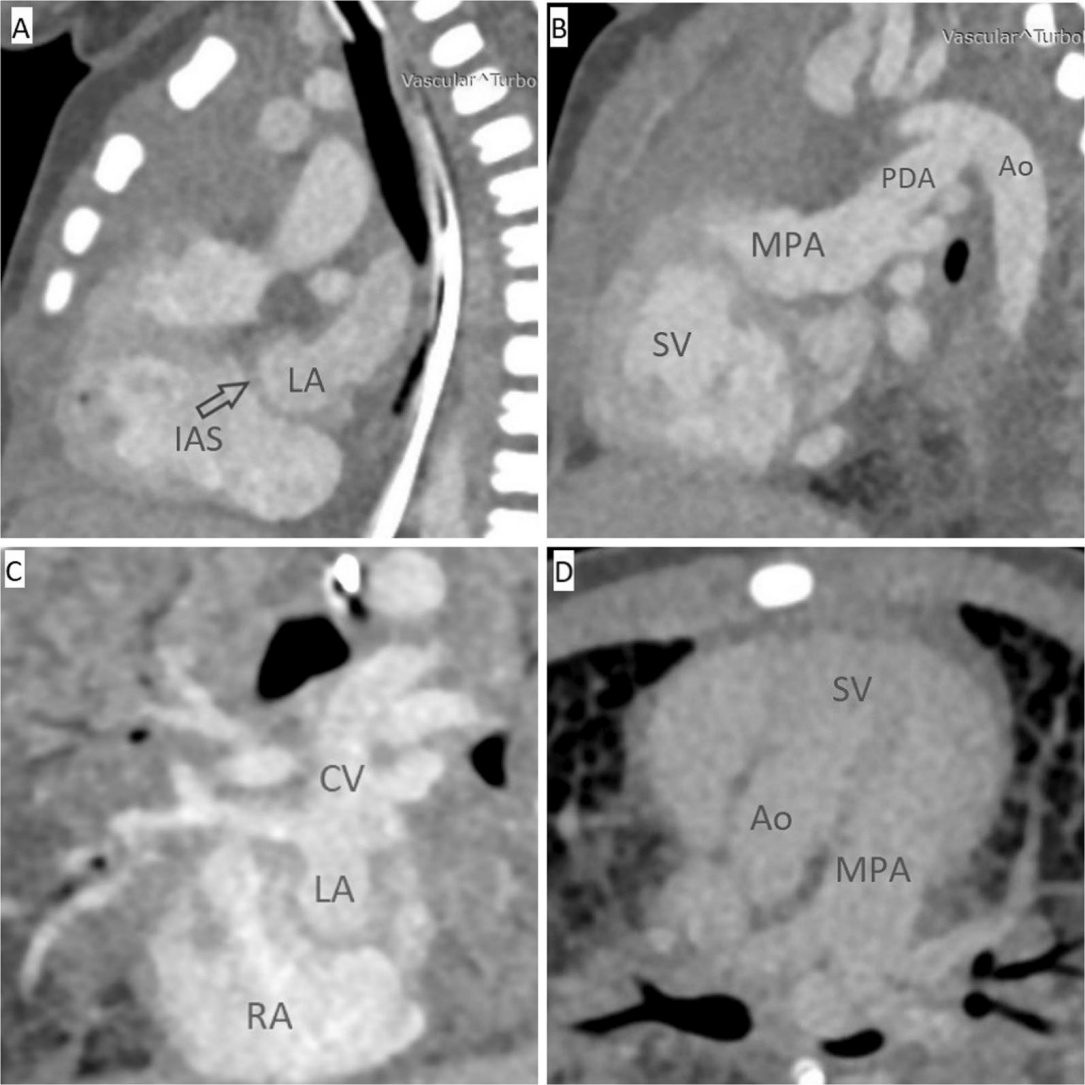
CT of chest. **A** Intact atrial septum (indicated by arrow). **B** Patent ductus arteriosus (indicated by arrow). **C** Cardinal vein (the only way outflow from the left atrium). **D** The aorta and pulmonary artery go from a single ventricle. *Ao* aorta, *CV* cardinal vein, *IAS* intact atrial septum, *LA* left atrium, *MPA* main pulmonary artery, *PDA* patent ductus arteriosus, *RA* right atrium, *SV* single ventricle

Considering the serious condition of the patient and the increasing respiratory failure, the child was transferred to a ventilator and transported to the radiology department for a computed tomography (CT) study to clarify the anatomy of the CHD. Computed tomography showed “MV atresia, left ventricular hypoplasia. Intact AS. DORV. Right-sided aorta, distal aortic arch hypoplasia, patent ductus arteriosus. Persistent cardinal vein. Obstruction of the cardinal vein” (Fig. 2).

**Fig. 3:**
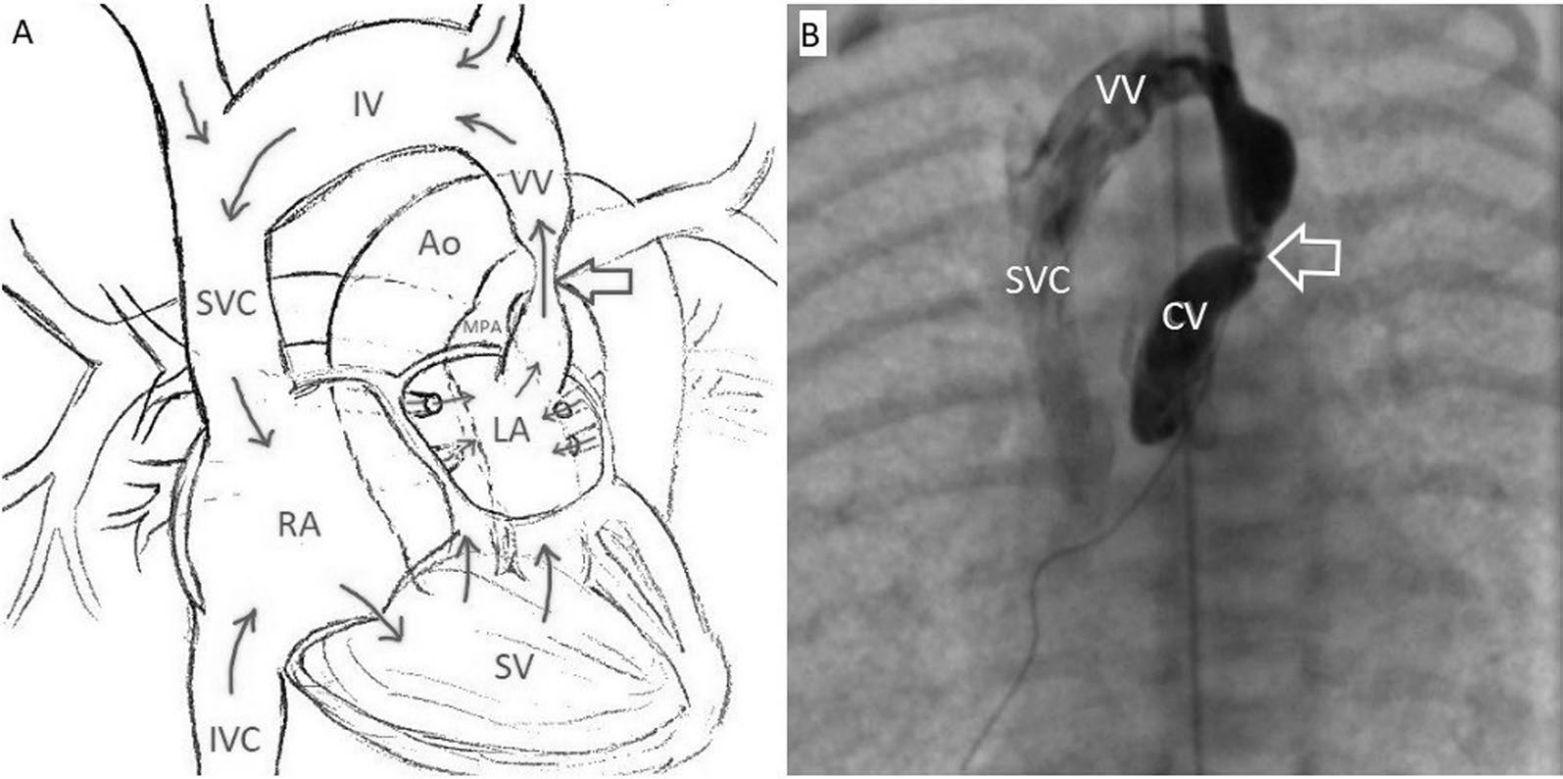
Anatomical representation of venous return anomaly at the primary form. **A** Schematic designation of venous outflow from the LA through a stenotic CV. **B** Angiography with stenosis zone visualization (indicated by the arrow) of the CV, anteroposterior view. *Ao* aorta, *CV* cardinal vein, *IV* innominate vein, *IVC* inferior vena cava, *LA* left atrium, *MPA* main pulmonary artery, *RA* right atrium, *SV* single ventricle, *SVC* superior vena cava

Considering the progression of the patient’s cardiopulmonary failure, desaturation reaching 75% (PaO_2_ = 67.4 mmHg at FiO_2_ 90%), and lactic acidosis reaching 5.2 mmol/l, endovascular stenting of the narrowed section of the cardinal vein was conducted to stop the pulmonary edema and patient’s condition. One hour after birth, the child was taken into the operating room of the Department of X-ray Surgical Methods of Diagnosis and Treatment, where the left internal jugular vein was catheterized, and a 6 Fr introducer was installed. Next, the coronary conductor "Fielder" 0.014' was passed through the stenotic area of ​​the cardinal vein into the left atrium (LA) cavity. A diagnostic "multipurpose" (5Fr) catheter was passed along the guidewire to the CV, and angiography was performed (Fig. 3).

**Fig. 4:**
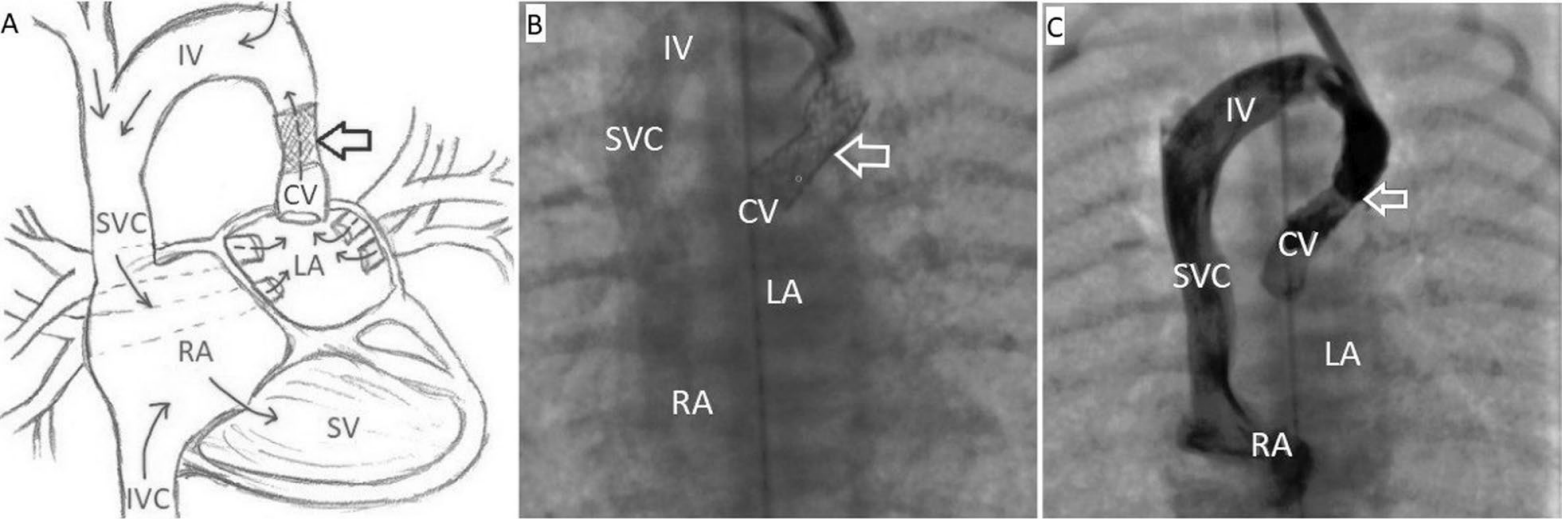
Anatomical representation of venous return anomaly after cardinal vein stenting. **A** Schematic representation of venous outflow from the left atrium after cardinal vein stenting. **B** The stent is implanted in the narrowed area (indicated by the arrow), anteroposterior view. **C** The area of ​​stenting (indicated by the arrow) is passable, the contrast agent freely enters the innominate vein, anteroposterior view. *CV* cardinal vein, *IV* innominate vein, *IVC* inferior vena cava, *LA* left atrium, *RA* right atrium, *SV* single ventricle; SVC, superior vena cava

Stenosis of the proximal segment of the cardinal vein with narrowing up to 1.5 mm was confirmed (proximal and distal stenosis of the vein up to 6 mm in size). Then, the drug-eluting stent (DES) "Dynamic Renal" 6.0 × 15 mm (*p* = 8 atm) was positioned and implanted along the guidewire in the stenotic area of ​​the cardinal vein. Control angiography was performed: the stent was positioned with Doppler ultrasonography, and the flow size was up to 5.3 mm (Fig. 4).

There was an increase in saturation from 70 to 85% within 5 min after stent implantation. The patient was transferred to the intensive care unit for children under the supervision of a resuscitator for cardiac surgery pathology.

**Fig. 5:**
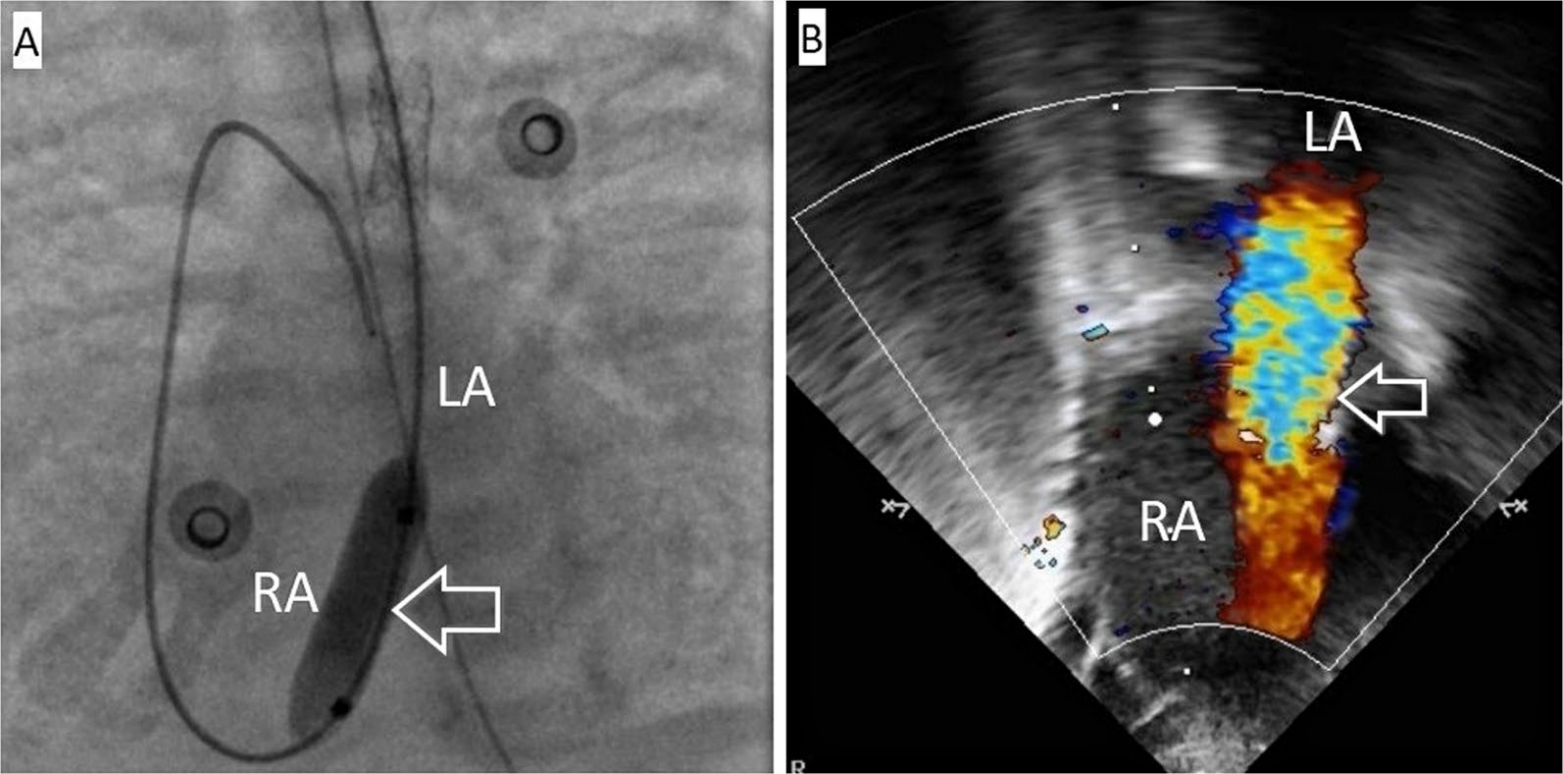
Balloon dilatation of the AS under simultaneous X-ray and Echocardiography. **A** Balloon septostomy under angiographic control, the balloon (indicated by the arrow) is inflated in the area of ​​the atrial septal defect, anteroposterior view. **B** Echocardiography after balloon septostomy. Interatrial communication is 3 mm in size (indicated by an arrow). *LA* left atrium, *RA* right atrium

In the immediate postoperative period, positive dynamics were observed in the normalization of hemodynamic and respiratory parameters (Sat 85% with FiO_2_ 30%). However, over the next week, signs of congestion in the lungs gradually increased. Despite the increase in ventilator parameters, PvO_2_ varied in the range of 30–35 mmHg, PvCO_2_ was approximately 50–58 mmHg, and blood lactate levels reached 4.4 mmol/l. An X-ray revealed severe pulmonary hypervolemia. ECHO data revealed restenosis of the outflow tract from the pulmonary venous collector. Due to the increase in signs of pulmonary venous hypertension and the increase in the pressure gradient in the area of ​​the cardinal vein stent, the child was urgently transported to the X-ray operating room. During the manipulation, the left internal jugular vein was punctured, a 5Fr introducer was installed, and the diagnostic catheter "multipurpose" (5Fr) was passed along the guidewire 0.035' to the stented section of the cardinal vein. Angiography was performed, and the restenosis of the stented section of the cardinal vein in the middle third of the stent reached 4.5 mm (proximal and distal to the site of stenosis, the vein was up to 6.0 mm in size). The 5Fr introducer was replaced with the 6 Fr introducer. Then, the balloon catheters "Quantum Maverick" 5.0 × 15 mm and "Sterling" 5.5 × 20 mm were passed alternately to the stenotic area. Balloon angioplasty was performed, and the deformed section of the stent expanded at a pressure in the balloon of 12 atm. Despite the complete expansion of the stent up to 5.5 mm, control echocardiography showed a high speed in the stented area (up to 3.0 m/s). The mean invasive pressure in the left atrium was approximately 28 mmHg. The decision was made to perforate the atrial septum. Under simultaneous navigation by echocardiography and radiography, considering the anatomically advantageous position of the cardinal vein, it was possible to pass the catheter to the interatrial septum so that the catheter tip rested and stretched the central part of the atrial septum. Furthermore, a guidewire 0.035' was passed along the catheter, and the septum was perforated. The conductor was placed in the right atrium cavity. Then, a balloon catheter ("Admiral Xtreme", 7.0 × 20 mm) was delivered through the guidewire to the area of ​​the interatrial communication, and dilatation of the AS was performed (Fig. 5).

**Fig. 6:**
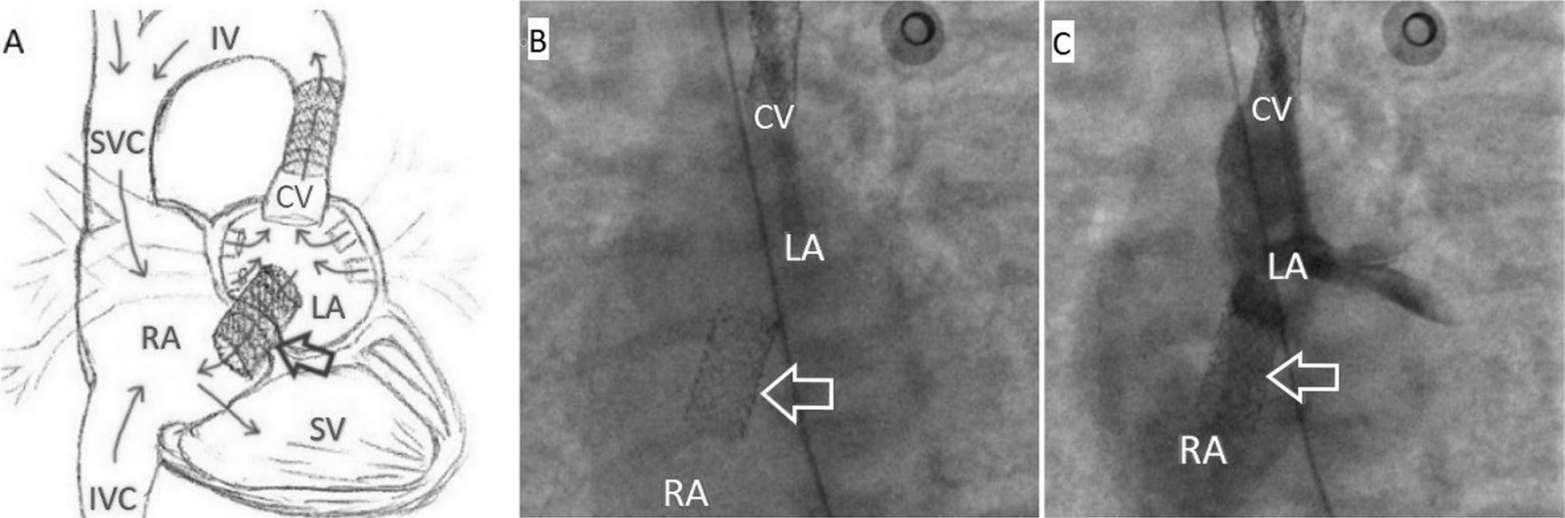
Anatomical representation of venous return abnormality after stenting of the CV and interatrial communication. **A** Schematic illustration of venous outflow from the LA after stenting of the CV and interatrial communication. **B** Stent is in the area of ​​the AS (indicated by an arrow), anteroposterior view. **C** The area of ​​stenting is passable, the contrast from the LA flows into the RA through the stented CV and the stented interatrial communication (indicated by the arrow), anteroposterior view. *CV* cardinal vein, *IV* innominate vein, *IVC* inferior vena cava, *LA* left atrium, *RA* right atrium, *SV* single ventricle, *SVC* superior vena cava

The ECHO data revealed that the flow through the created interatrial communication reached 3 mm, while the pressure in the LA remained high (mean pressure was approximately 28–30 mmHg). A decision was made to stent the AS. Then, a DES "Dynamic Renal" stent 7.0 × 15 mm (*p* = 12 atm) was positioned and implanted in the area of ​​the interatrial communication. On ECHO and control angiography, the flow through the stented interatrial communication reached 6.0–7.0 mm (Fig. 6).

After AS stenting, the mean pressure in the right atrium (RA) was 14 mmHg, and the mean pressure in LA decreased to 16 mmHg. There was an increase in saturation from 70 to 95% (at FiO_2_ 40%). The patient was transferred to the cardiac intensive care unit.

Subsequent control echocardiography showed no obstruction on the stents. An X-ray examination revealed positive lung dynamics in the form of a significant recovery of pneumatization of the lung tissue. The child's condition stabilized, and there was a positive trend of laboratory parameters in the form of decreasing PvCO_2_ up to 40–45 mmHg and the blood lactate level up to 1.3 mmol/l. However, due to the absence of subpulmonary obstruction, signs of high pulmonary hypertension persisted. Thus, we could not delay the next stage of the treatment. Three days later, the newborn underwent excision of the atrial septum with stent removal, ligation and transection of the cardinal vein with stent removal, ligation of the patent ductus arteriosus and pulmonary artery trunk narrowing by a cuff made of a “Gore-Tex” vascular prosthesis using cardiopulmonary bypass. The ECHO data revealed that the size of the pulmonary trunk at the cuff level reached 5.5 mm (flow velocity was 4.5 m/s). The gradient at the cuff level was 30–35 mmHg. The child was transferred to the intensive care unit. The postoperative period proceeded without complications. The duration of the patient's stay in the intensive care unit (ICU) was 36 days, and inotropic therapy and artificial lung ventilation was conducted for 32 days. After condition stabilization in the ICU, the child was transferred to the Department of Pathology of Newborns and Premature Infants for further observation. Saturation at rest was 78–85%, and weight gain and height increases correlated with age. The patient was discharged from the hospital and rehospitalized in a planned manner 4 months later for the next stage of CHD correction, i.e., the imposition of a bidirectional cavopulmonary anastomosis (BCPA, Glenn procedure). The postoperative period was smooth. The child is currently being observed by a pediatrician at their place of residence. The patient’s condition is stable without negative dynamics, and blood oxygen saturation is approximately 80–85%.

## Conclusions

Congenital anomalies of pulmonary venous return represent a large group of CHDs. One of the critical aspects of pulmonary vein pathology is obstruction, which determines the clinical course of the disease [7, 8]. The clinical presentation of pulmonary vein obstruction may be associated with both anatomic narrowing of the pulmonary veins proper (e.g., congenital primary pulmonary vein stenosis) and narrowing of drainage pathways directly or indirectly associated with the pulmonary veins (e.g., vertical vein stenosis in TAPVC).

Obstructed pulmonary venous blood flow is one of the main indications for surgical correction of the defect as soon as possible [3, 4]. In addition, unstable hemodynamics, the development of respiratory distress syndrome, and the combination of this defect with other CHDs, for example, with a SV, increase the risk of postoperative mortality, which, according to the literature, occurs in 41.7–53% of patients [9, 10].

In this article, we presented the clinical case of a patient with a SV, DORV, MV atresia, and an intact AS. One feature of this case was the presence of a stenotic persistent cardinal vein, through which blood from the LA flowed into the innominate and further into the SVC. Considering the anatomical features of the pulmonary venous blood flow drainage pathways, increasing signs of tissue hypoxia in the form of an increase in the blood lactate concentration along with extremely severe hemodynamic disorders requiring inotropic support, the risk of open surgery remained high. Therefore, as a primary operation, palliative endovascular treatment was performed by stenting the stenotic section of the cardinal vein to alleviate the manifestations of acute pulmonary venous obstruction. Despite the successful surgical intervention and improvement in clinical and laboratory parameters, signs of systemic venous return obstruction persisted, necessitating a second minimally invasive procedure to stent the atrial septum. After stabilization of the child's condition, narrowing of the pulmonary artery trunk was performed, followed by the imposition of a BCPA. Thus, transcatheter intervention in infants with an obstructive form of venous return anomaly in combination with a univentricular CHD can be considered a new concept within a staged approach to treatment, stabilizing the patient’s condition before open surgical correction.

## Data Availability

The datasets used and/or analyzed during the current study are available from the corresponding author on reasonable request.

## Abbreviations

AS: Atrial septum
BCPA: Bidirectional cavopulmonary anastomosis
CHD: Congenital heart defect
CT: Computed tomography
CV: Cardinal vein
DES: Drug-eluting stent
DORV: Double outlet right ventricle
ECHO: Echocardiography
ICU: Intensive care unit
IV: Innominate vein
LA: Left atrium
MV: Mitral valve
PV: Pulmonary vein
RA: Right atrium
SV: Single ventricle
SVC: Superior vena cava
TAPVC: Total anomalous pulmonary venous connection
